# Differential Modulation of Innate Antiviral Profiles in the Intestinal Lamina Propria Cells of Chickens Infected with Infectious Bursal Disease Viruses of Different Virulence

**DOI:** 10.3390/v14020393

**Published:** 2022-02-15

**Authors:** Rui Chen, Jinnan Chen, Yanhua Xiang, Yanyan Chen, Weiwei Shen, Weiwei Wang, Yihai Li, Ping Wei, Xiumiao He

**Affiliations:** 1 Guangxi Key Laboratory for Polysaccharide Materials and Modifications, School of Marine Sciences and Biotechnology, Guangxi University for Nationalities, Nanning 530008, China; cr17635489890@163.com (R.C.); jinnanchen123@sina.com (J.C.); xyh2485631921@163.com (Y.X.); cyy15777136876@163.com (Y.C.); shenww2022@163.com (W.S.); hornets@163.com (Y.L.); 2 Institute for Poultry Science and Health, Guangxi University, Nanning 530004, China; wangweiweihn@163.com

**Keywords:** infectious bursal disease virus, gut-associated lymphoid tissues (GALT), intestinal lamina propria (ILP) cells, innate antiviral response, reverse transcription quantitative real-time PCR (RT-qPCR)

## Abstract

Infectious bursal disease virus (IBDV) is one of the most important infectious diseases of poultry around the world. Gut-associated lymphoid tissues (GALT) are the first line of defense of the host against the infection. The purpose of this study was to investigate the role of innate immune antiviral signaling triggered by Toll-like receptor 3 (TLR3), as well as macrophage activation and cytokine response in the intestinal lamina propria (ILP) cells after the oral challenge of IBDV in relation to IBDV virulence and disease pathogenesis. The results showed that the expression levels of TLR3, IRF7, IFN-α/β and the corresponding downstream antiviral factors OAS, PKR and Mx were all upregulated in the SPF chicken ILP cells at 8 h post-infection (hpi) and 12 hpi. Similarly, macrophages were activated, with the initial macrophage M1 activation observed at 8 hpi, but then it rapidly shifted to a non-protective M2-type. Both Th1 (IFN-γ, TNF-α, IL-12) and Th2 (IL-4 and IL-10) types of cytokines were differentially upregulated during the early stage of infection; however, the Th1 cytokines exhibited stronger activation before 8 hpi compared to those of the Th2 cytokines. Interestingly, differential regulations of gene expression induced by different IBDV strains with different virulence were detected. The HLJ0504-like very virulent (vv) IBDV strain NN1172 induced stronger activation of TLR3-IFN-α/β pathway, macrophages and the Th1/2 cytokines’ expression, compared to those induced by the attenuated strain B87 at 8 hpi and 12 hpi in the ILP cells. In conclusion, the innate antiviral response mediated by the TLR3-IRF7 pathway, macrophage activation and cytokine expression in the GALT cells at the early stage of IBDV infection was differentially modulated, and the HLJ0504-like vvIBDV strain triggered stronger activation than the attenuated vaccine strain, and that may play an important role in the progression of disease.

## 1. Introduction

Infectious bursal disease (IBD), which is caused by infectious bursal disease virus (IBDV), is an acute, highly contagious and immunosuppressive viral disease in poultry, and threatens the poultry industry throughout the world [1]. IBDV is a member of *Avibirnavirus* of the family *Birnaviridae*, and is characterized by destruction of lymphoid organs in 3 to 6-week-old chickens. Two serotypes of IBDV (serotype 1 and serotype 2) are reported, and only serotype 1 is pathogenic to chickens, with five pathotypes with an increasing order of virulence from mild (attenuated), intermediate, classical, antigenic variants (av), to very virulent (vv) [2]. vvIBDV can break through the protection of high titers of a maternally derived antibody and cause high rates of mortality in young birds [3]. Although many inactivated and attenuated live vaccines are used to control the disease, the emergence of vv, av and reassortant IBDV strains in recent years makes the prevention and control of IBD ever more complicated [4,5,6,7].

Gut-associated lymphoid tissues (GALT) are the first line of defense of the host against the oral infection of IBDV, after which the virus replicates in macrophages and lymphoid cells of the gut, and then enters the portal circulation, leading to primary viraemia [8]. The chicken GALT is organized in caecal tonsils (CT), Peyer’s patches (PP), Meckel’s diverticulum and other lymphoid aggregates located within the lamina propria (LP) along the gastrointestinal tract [9,10], which all contribute to local defense against invading pathogens as well as to the systemic immune responses [11]. However, apart from the bursa of Fabricius (BF), limited information is available about the effects of IBDV on the GALT and what its role is on the pathogenesis of IBDV. Müller et al. [12] and Saif [13] reported that lymphocytes and macrophages in the intestine played a role in vvIBDV transmission to the BF and other sites. Wang et al. [14] demonstrated that vvIBDV infection led to a decrease in villus height, and a reduction in the number of intestinal intraepithelial lymphocytes (IEL) and mast cells in the intestine of specific-pathogen-free (SPF) chickens. Recently, Li et al. [9] further confirmed the rapid replication of vvIBDV in the CT and caecum, demonstrating that vvIBDV infection had a rapid (during the first 72 h) and significant impact on the GALT and the gut microbiota composition, which in turn could explain increased susceptibility of the infected birds to secondary pathogens.

The outcome of a viral infection is partly mediated through the complex interplay of viral and cellular innate immune responses, especially those mediated by type 1 interferons. During virus entry and replication, the innate antiviral immune response machinery senses the invading virus and activates a cascade of antiviral signaling pathways to create a viral replication-restricting intracellular environment [15,16]. In turn, viruses evolved strategies to disable the host’s innate antiviral immune arsenal and optimize the intracellular environment for their efficient replication and release. Studies suggested that viral interference with the TLR3-IFN-β innate pathway in the BF plays an important role in the pathogenesis of IBDV [17,18], while Liu et al. [19] showed that some of the Th1 and Th2 cytokines were differentially upregulated after vvIBDV infection. However, these relations were not well studied in the GALT. Our objective was to investigate the role of innate immune antiviral signaling triggered by TLR3, macrophage activation and cytokine expression in the intestinal lamina propria (ILP) at the early stage of infection on the pathogenicity of IBDV strains with different virulence.

## 2. Materials and Methods

### 2.1. Viruses

The virus strain NN1172 (HLJ0504-like vvIBDV) was a field strain isolated and identified by our group [4,20]. The commonly used commercial attenuated live vaccine strain B87 with intermediate virulence was purchased from HLJ Animal-use Biological Products Co., Ltd., Beijing, China. All the viruses were propagated in 9-day-old specific pathogen free (SPF) embryonated chicken eggs and titrated using the methods previously described [21]. The titer of virus used for infection was 10^4^ EID_50_ per 0.2 mL per chicken.

### 2.2. Chicken Embryos and Chickens

SPF embryonated chicken eggs and four-week-old SPF chickens were purchased from Beijing Merial Vital Laboratory Animal Technology Co., Ltd., Beijing, China. All the SPF chickens were transferred to the isolation facility, and no vaccination was administered.

### 2.3. Experimental Design

In order to test the abilities of IBDV modulating the expression of the chicken innate antiviral pathway, including TLR3, IRF7, IFN-α/β and the downstream antiviral proteins PKR, OAS, and Mx at the early infection stage in the gut, eighty-five 4-week-old SPF chickens were divided into 3 groups as described in Table 1. Thirty-five birds in group 1 were infected with the IBDV field strain NN1172, and 15 birds in group 2 were infected with the vaccine strain B87. All birds in groups 1 and 2 received orally a titer of 10^4^ EID_50_/0.2 mL viruses, respectively. Thirty-five birds in group 3 were served as a mock-infection control. The intestinal tissues were harvested at different time points during the first 12 h post-infection (hpi) and detected the following: duodenum and cecal tonsil samples from 5 birds in groups 1 and 3 were harvested, respectively, and detected by immunohistochemistry for the IBDV antigen using the monoclonal antibody at 4, 8 and 12 hpi; then, the other parts of the small intestinal in each bird from all the groups were collected for the isolation of ILP cells for the detection of the innate antiviral response, the cytokine production and the phenotypes of macrophages, respectively. In group 1, the viral load in the ILP cells at 4 hpi, 8 hpi and 12 hpi and the BF at 1, 3 and 5 days post infection (dpi) were further detected (*n* = 5). The bursa/body-weight index (BBIX) ratio was also determined at 1, 3 and 7 dpi (*n* = 5). A BBIX less than 0.70 was considered as atrophy [22]. The antiviral and cytokine response in groups 1 and 2 were compared for evaluating the differential expressions of TLR3-IRF7-IFN-β, macrophage activation and cytokines’ response induced by the IBDV strains with different virulence in birds at the early stage in the gut. The viral load and the expressions of TLR3-IRF7-IFN-β, cytokines, nitric oxide synthase (iNOS) and Arg (Arginase) were detected by RT-qPCR.

### 2.4. Immunohistochemical Detection of the IBDV Antigen

In order to detect whether the IBDV colonized in the ILP cells at the early stage, the duodenum and cecal tonsil from the infected birds were collected. Sections of the cecal tonsils and duodenum were then fixed in 10% formalin solution and prepared as previously described [9]. The IBDV antigen was detected using a monoclonal anti-IBDV-VP2 antibody (a kind gift from Professor Aijian Qin, Yangzhou University, Yangzhou, Jiangsu, China) at a dilution of 1:10,000. The secondary anti-mouse IgG HRP-labeled antibodies were used according to the manufacturer’s instructions. DAB (DAB peroxidase substrate Kit, ZSGB-bio, Beijing, China) was used to visualize the enzyme-linked complex. Sections were observed by light microscopy.

### 2.5. Isolation of ILP Cells of Birds

The ILP cells were isolated from the small intestines according to methods described by Davis and Parrott [23] and Sfeir et al. [24]. Briefly, the small intestines were removed from birds and thoroughly washed with cold PBS using a 50 mL syringe fitted with a short plastic cannula to remove all food residuals and mucus. All blood vessels, fat and mesentery were completely removed from the cleaned small intestines. The intestines were then opened longitudinally and cut laterally into small pieces (1–3 cm in length). These slices were washed extensively in cold PBS, transferred to flasks containing 10 mL of dissociation solution (NaCl 8 g, KCl 0.4 g, Na_2_HPO_4_·12H_2_O 0.121 g, KH_2_PO_4_ 0.06 g, NaHCO_3_ 0.35 g, glucose 1 g, 2 mM EDTA, 1 mM DTT; add ddH_2_O to final volume of 1 L, pH 7.4) and incubated at 37 °C for 30 min with stirring. The EDTA in this step could strip off the epithelial cells, and the left cells are ILP cells. The slices were then washed with PBS 2–3 times and then incubated for 30 min with stirring in 10 mL RPMI medium containing 200 U/mL collagenase. After incubation, 1 mL RPMI medium containing 10% fetal serum was added to the solution to stop the cell digestion. Finally, the gut slices were pumped slowly up and down through a 5 mL syringe to facilitate total disruption. The resultant ‘soup’ was then filtered through a 40 µm filter and washed twice with RPMI. Final cell viability, as assessed by the trypan blue dye exclusion test, was higher than 90% for all samples. The cells were finally suspended in RPMI medium to a concentration of 10^6^ cells/mL, and 1 mL of which was used for RNA isolation.

### 2.6. Reverse Transcription Real-Time Quantitative PCR (RT-qPCR)

ILP cells or BF samples collected from birds of the infected and uninfected control group were subjected to RNA extraction by using TRIzol reagent (CWBIO, Beijing, China) according to the manufacturer’s instructions. Briefly, 10^6^ cells were suspended in 1 mL of TRIzol reagent, and the total RNA was prepared according to the manufacturer’s instructions. Finally, total RNA extracted from each sample was suspended in 30 μL of RNase-free water. For viral RNA extraction, the cells or BF samples (after homogenization) were frozen and thawed 3 times and centrifuged at 3000× *g* for 10 min, and 200 μL of the supernatants were used for RNA isolation. Then, 1 μg of the RNA sample was used for cDNA synthesis using the HiFiScript 1st Strand cDNA Synthesis Kit (CWBIO, Beijing, China) according to the manufacturer’s instructions. The contamination of genome DNA was excluded by the gDNA wiper (CWBIO, Beijing, China).

RT-qPCR was performed on cDNA using SYBR Premix Ex Taq II (TAKARA, Dalian, China) in a Step One Plus (ABI, Waltham, MA, USA). The protocol for the detection of the transcription level of chicken TLR3, IRF7, IL-4, IL-10, IL-12, IFN-α/β, IFN-γ, OAS, PKR, Mx and GAPDH was previously established, and the primers are listed in Table 2. The protocol for the detecting of iNOS, Arg and TNF-α was established in this study. Briefly, the primers (Table 2) were designed using Primer 5.0 software (Primer-E Ltd., Plymouth, UK). The vector pUC-T (CWBIO, Beijing, China) carrying the iNOS, Arg and TNF-α, respectively, was serially diluted ten-fold and used to generate a standard curve. The R^2^ of the calibration curve and Amplification efficiency (E) were also evaluated, and the results are shown in Supplementary Table S1. The established reaction mixture (20 μL), containing 10 μL SYBR Premix Ex Taq II, 10 μM forward primer, 10 μM reverse primer, 2 μL cDNA and 0.4 μL ROX Reference Dye, was subjected to the following thermal cycling procedure: 95 °C for 30 s, 40 cycles of 95 °C for 5 s and 58 °C (iNOS), or 55 °C (Arg and TNF-α) for 30 s. The melting curve analysis after amplification was performed from 65 °C to 95 °C with a ramp speed of 1 °C/s. The relative expression of the target gene was normalized to GAPDH mRNA using the 2^−ΔCt^ method, where ΔCt = Cttarget gene − CtGAPDH. The fold changes for each gene were calculated as the expression level of the gene in the infected group versus that in the uninfected group.

The RT-qPCR protocol for the detecting of viral load based on the IBDV VP2 gene was previously established by our group [31].

### 2.7. Statistical Analysis

Statistical significance was calculated using the Student’s *t*-test for individual paired comparisons or one-way ANOVA whenever multiple groups were compared. For individual comparisons of multiple groups, the Student–Newman–Keuls post-hoc test was used to calculate *p* values. All values are reported as means ± standard errors (SEM). All statistical calculations were performed using Primer of Biostatistics [32].

### 2.8. Ethical Statement

Animal experiments in this study were performed in accordance with the protocols of Animal Welfare and approved by the Animal Experimental Ethical Committee of Guangxi University for Nationalities, and the approval code is 202110608004.

## 3. Results

### 3.1. Colonization of IBDV in ILP Cells and the Replication of IBDV in BF

**Figure 1:**
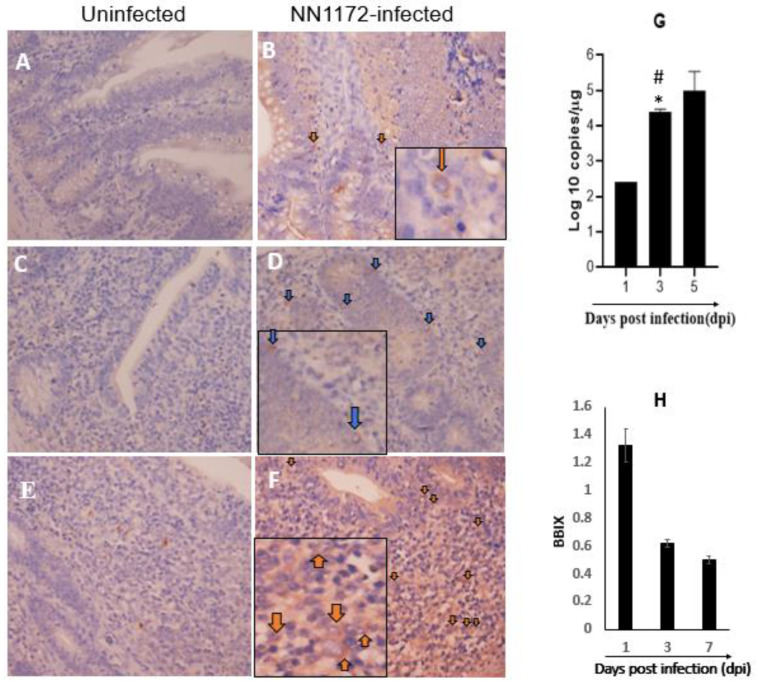
The expression of IBDV-VP2 protein in the ILP cells and the viral load in the BF in NN1172-infected birds. (**A**–**F**), Immunohistochemistry results obtained from the duodenum and cecal tonsil. The brown-yellow ring staining around the nucleus in the cytoplasm of the cells was regarded as the IBDV antigen positive and indicated with arrow. (**A**) uninfected duodenum at 4 hpi which showed negative for IBDV; (**B**) IBDV-infected duodenum at 4 hpi which showed positive for IBDV; uninfected cecal tonsil at 4 hpi (**C**) and 9 hpi (**E**) which showed negative for IBDV; IBDV-infected cecal tonsil at 4 hpi (**D**) and 9 hpi (**F**) showed positive for IBDV. (**G**) The viral load in the BF at 1, 3, 5 dpi of IBDV infection. The viral RNA copies were normalized to μg of RNA sample. Note: * *p* < 0.05 between 3 dpi and 5 dpi. # *p* < 0.05 between 1 dpi and 3 dpi; (**H**) The BBIX value of the IBDV-infected birds at 1, 3 and 7 dpi. *n* = 5. Note that the BF of the infected chicken was atrophic at 3 and 7 dpi.

To determine the colonization and replication of the IBDV in the ILP cells, we quantified the IBDV antigen by immunohistochemistry (Figure 1A–F). A positive signal could be first detected at ILP cells in the duodenum (Figure 1B) and cecal tonsil as early as 4 hpi (Figure 1D); significant IBDV antigen positive signals were detected in the cecal tonsil at 9 hpi (Figure 1F). No IBDV antigen was detected in the mock-infected birds at all the time points examined (Figure 1A,C,E). The results of the quantification detection of IBDV infection showed that the viral load in ILP cells varied over times of infection, which was demonstrated by the data that both the IBDV-positive and IBDV-negative results were obtained (data not shown), as shown at the indicated time points of 4, 8 and 12 hpi. However, increasing viral load was observed in the BF at 1, 3 and 5 dpi (Figure 1G). The BBIX evaluation showed that the BF was atrophic in the infected birds at 3 and 7 dpi, as evidenced by the BBIX value being lower than 0.7 (Figure 1H), consistent with the result of our group previously reported [20,33]. These results indicated that IBDV colonized in the ILP cells and replicated in the infected birds.

### 3.2. Upregulation of the Innate Antiviral Response in ILP Cells

**Figure 2:**
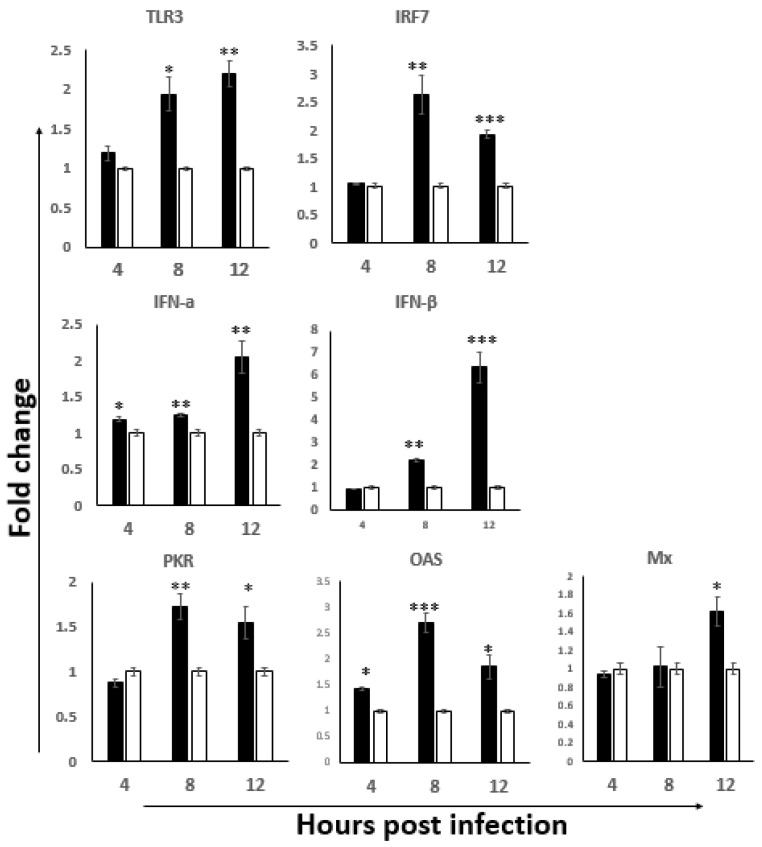
The gene expression level of TLR3 antiviral pathway in the ILP cells of NN1172 infected birds. The mRNA expression levels of TLR3, IRF7, IFN-α, IFN-β, PKR, OAS and Mx were detected at 4, 8 and 12 hpi. Results represent mean ± SEM, *n* = 5. (* *p* < 0.05; ** *p* < 0.01; *** *p* < 0.001) between the infected-group (black bar) and the uninfected-group (white bar).

To investigate whether IBDV infection activates the innate antiviral response in ILP cells, the mRNA expression levels of the TLR3-IRF7-IFN-α/β and the downstream antiviral factors PKR, OAS and Mx were detected by RT-qPCR in the total RNA extracted from ILP cells. As shown in Figure 2, the mRNA expression levels of TLR3, IRF7, IFN-α/β were all upregulated at 8 hpi and 12 hpi, as well as the downstream antiviral response factors. Among the earliest responses, we saw a significant upregulation of IFN-α and OAS, while most of the other factors were upregulated by 8 h, with IRF7, PKR and OAS reaching their peak response at this time. At 12 hpi, the expression of Mx became upregulated, and peak responses during the studied time course were noted for TLR3 and IFN-α/β. The results imply that the oral infection of IBDV results in a rapid activation of the TLR3 antiviral signaling pathway in ILP cells during the first hours of infection.

### 3.3. Activation of the Intestinal Macrophages

Macrophage activation, including classically activated macrophages (M1) and alternatively activated macrophages (M2), is a key step in the clearance of pathogenic agents. To assess if the M1 and M2 macrophages’ activation pathways were activated in the ILP cells, the transcriptional expressions of the M1 hallmark gene iNOS and the M2 hallmark gene Arg were detected; the iNOS/Arg ratio was also calculated as a readout of M1 vs. M2 bias to assess the polarization of the macrophage.

**Figure 3:**
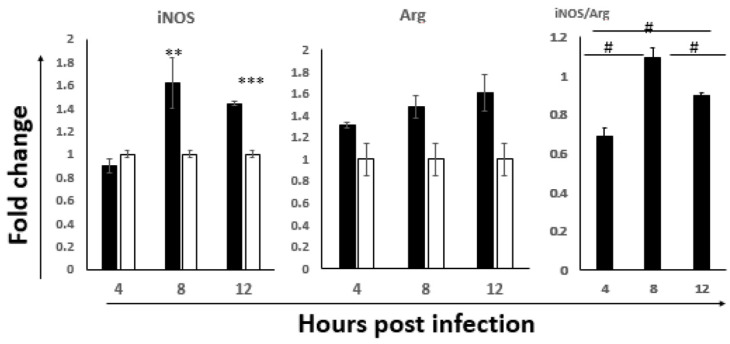
The expression of M1 and M2 hallmarks in the ILP cells of NN1172-infected birds at the early stage of infection. The mRNA expression levels of M1 phenotype Macrophage (iNOS) and M2 phenotype Macrophage (Arg) in the IBDV infected birds were detected at 4, 8 and 12 hpi. The iNOS/Arg ratio was calculated at each time point. Results represent mean ± SEM, *n* = 5. (** *p* < 0.01; *** *p* < 0.001) between the infected-group (black bar) and the uninfected-group (white bar). ^#^ *p* < 0.05 of iNOS/Arg ratio between each time point post-infection.

As shown in Figure 3, the iNOS expression level was significantly upregulated at 8 hpi and 12 hpi, peaking at 8 h. Interestingly, the level of the Arg gene also exhibited a trend of increase over time with the highest response at 12 hpi. The results suggested activation of macrophages in the ILP cells, especially M1 activation. Further, when the iNOS:Arg ratio was analyzed, our data showed a phenotype of M1 bias at 8 hpi, but then shifted back towards the M2 phenotype at 12 hpi. These results suggested that at the early stage of IBDV infection in the gut, initially they are M1 dominantly activated, but after 8 hpi, they start to revert to a non-protective M2 phenotype.

### 3.4. Enhanced Cytokine Response in ILP Cells

**Figure 4:**
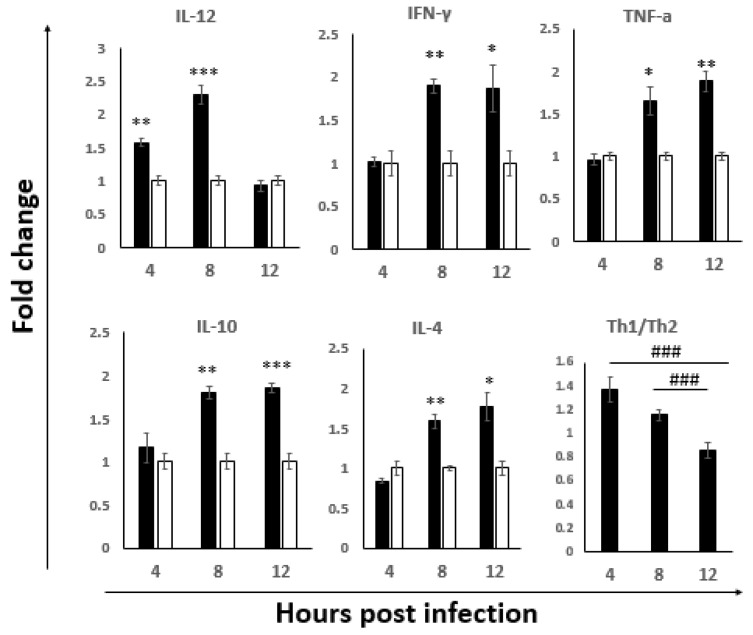
The expression of Th1 and Th2 cytokines in the ILP cells of NN1172-infected birds at early stage of infection. The mRNA expression levels of cytokines in the ILP cells of IBDV infected birds were detected at 4, 8 and 12 hpi. Th1/Th2 bias ratio was calculated as average of each Th1/Th2 cytokine. Results represent mean ± SEM, *n* = 5, (* *p* < 0.05; ** *p* < 0.01; *** *p* < 0.001) between the infected-group (black bar) and the uninfected-group (white bar). ^###^ *p* < 0.001 of Th1/Th2 between each time point post IBDV infection.

The evaluations of cytokine mRNA expression indicated that, both the Th1 (IFN-γ, TNF-α, IL-12) and the Th2 (IL-4 and IL-10) types of cytokines were differentially upregulated during the early stage of infection (Figure 4). The upregulation of Th1-driving IL-12 was observed at 4 hpi, peaking at 8 hpi, and then returning to baseline at 12 hpi, whereas IFN-γ and TNF-α were following with significant upregulation at 8 hpi and remaining high at 12 hpi. The expression patterns of pro-Th2 cytokines, IL-10 and IL-4 were similar to that of IFN-γ and TNF-α, as demonstrated by the similar upregulation trend at 8 hpi and 12 hpi. The Th1/Th2 bias was further evaluated by the expression of each Th1 and Th2 types of cytokines in the infected group at each time point. A stronger Th1 bias at the 4 hpi with the subsequent shift to Th2 at 8 hpi was observed and continued to become even more Th2 biased at 12 hpi. These results suggested that the cytokines in the ILP cells were upregulated at the early stage of infection, with the stronger Th1 bias before 8 hpi, which could help to eliminate the virus; subsequently it shifted to a non-protective Th2 bias.

### 3.5. Differential Regulation of Antiviral Pathway Modulated by IBDVs with Different Virulence

**Figure 5:**
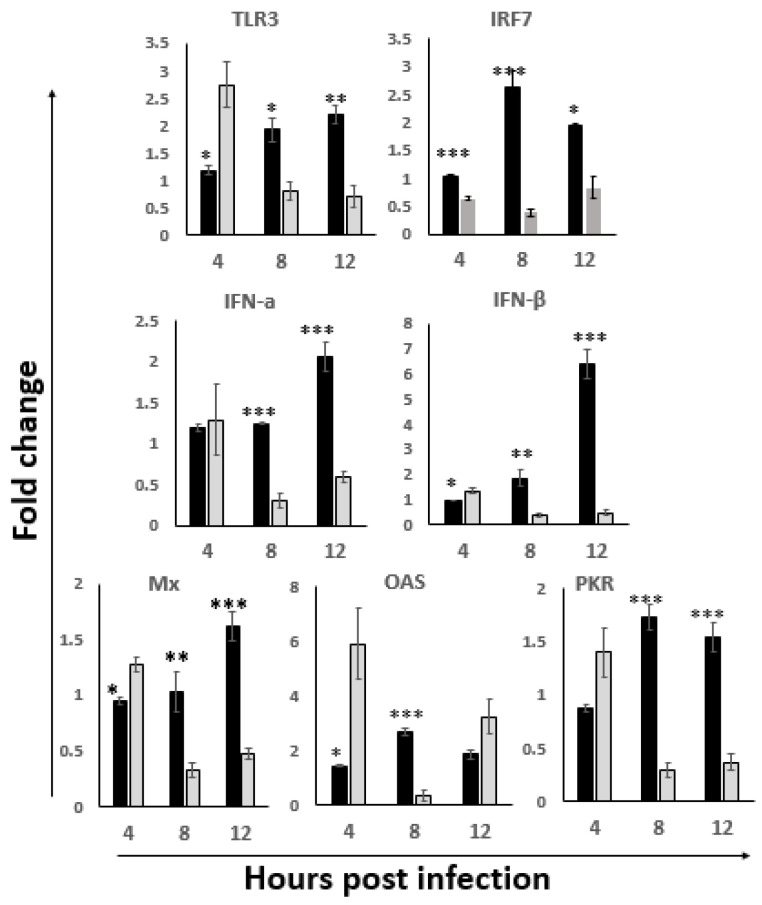
Differential modulation of TLR3 antiviral pathway in the ILP cells at the early stage of infection with IBDVs of different virulence. The mRNA expression levels of antiviral pathway genes in the ILP cells of IBDV infection were detected at 4 hpi, 8 hpi and 12 hpi. All the data were normalized to uninfected control. Results represent mean ± SEM, *n* = 5. (* *p* < 0.05; ** *p* < 0.01; *** *p* < 0.001) between the HLJ0504-like vvIBDV infected group (black bar) and the attenuated B87 infected group (gray bar).

The activation of the antiviral pathway in ILP cells correlated to the virulence of IBDV at the early stage of infection was further investigated by detecting the expressions of TLR3, IRF7, IFN-α/β, PKR, OAS and Mx in birds infected with the attenuated vaccine strain B87 and then compared with those in the birds infected with the HLJ0504-like vvIBDV strain NN1172. As shown in Figure 5, the NN1172 strain induced stronger activation of the antiviral signaling pathway than the B87 strain, as evidenced by the findings that a continuously higher level of expressions of TLR3, IRF7, IFN-α/β, Mx and PKR were detected in NN1172-infected birds at 8 hpi and 12 hpi, when compared to those detected in B87-infected birds. However, compared to the NN1172 strain, the B87 strain induced a relatively stronger activation of some molecules of the antiviral pathway at 4 hpi, including TLR3, IFN-β, Mx and OAS. Nevertheless, the expressions of IRF7, IFN-α and PKR were not significantly different between the two groups at 4 hpi. Interestingly, all these molecules were continuously downregulated at both 8 hpi and 12 hpi in the B87 group when compared to those in the NN1172 group. Further, the transcriptional expression of all the detected antiviral genes in the B87-infected chicken were similar to or less than the uninfected control as indicated by the fold change values (Figure 5). These results suggested that stronger activation of the innate antiviral response induced by the TLR3-IRF7-IFN-β pathway was observed in the NN1172 group than in the B87 group in the ILP cells at the early stage of IBDV infection.

### 3.6. Differential Modulations of the Macrophage and Cytokine Response by Different Virulent IBDV Strains in ILP Cells

**Figure 6:**
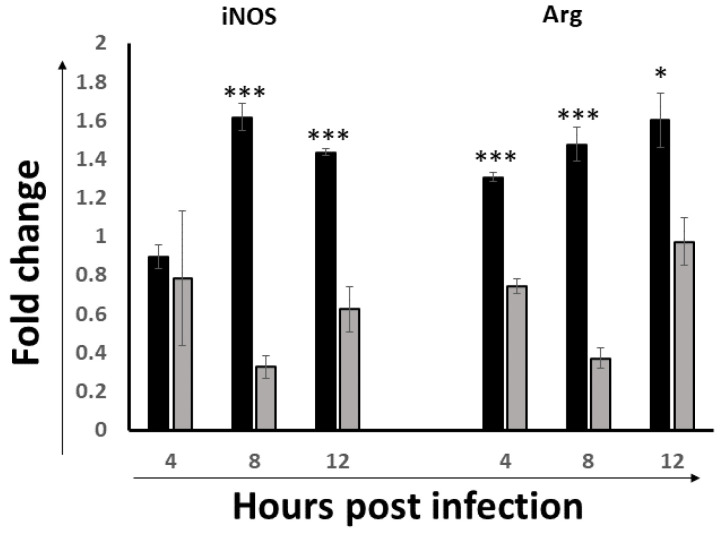
Differential modulations of M1 and M2 hallmarks in the ILP cells at the early stage of infection with IBDVs of different virulence. The mRNA expression levels of M1 phenotype Macrophage (iNOS) and M2 phynotype Macrophage (Arg) in the IBDV infected birds were detected at 4, 8 and 12 hpi. All the data were normalized to uninfected control. Note that higher activations of Macrophages in NN1172 infected birds than B87 infected birds at 8 hpi and 12 hpi. Results represent mean ± SEM, *n* = 5. (* *p* < 0.05; *** *p* < 0.001) between the HLJ0504-like vvIBDV infected group (black bar) and the attenuated B87 infected group (gray bar).

To understand whether the modulation of the macrophage and cytokine response correlated or not with the virulence of IBDVs, the hallmarks of macrophage and Th1/2 cytokines in the ILP cells were detected in the early stage of B87 infection and then compared with those in the NN1172 group. Results show that there was no difference in the expression levels of both the M1 (iNOS) and M2 (Arg) hallmarks between the NN1172 and B87 infected groups at 4 hpi, (Figure 6); however, significantly higher expression levels of iNOS and Arg in the NN1172 group were detected at 8 hpi when compared to those in the B87 group (*p* < 0.001). At 12 hpi, the activation of the M2 macrophage was still stronger in the NN1172 group than in the B87 group.

**Figure 7:**
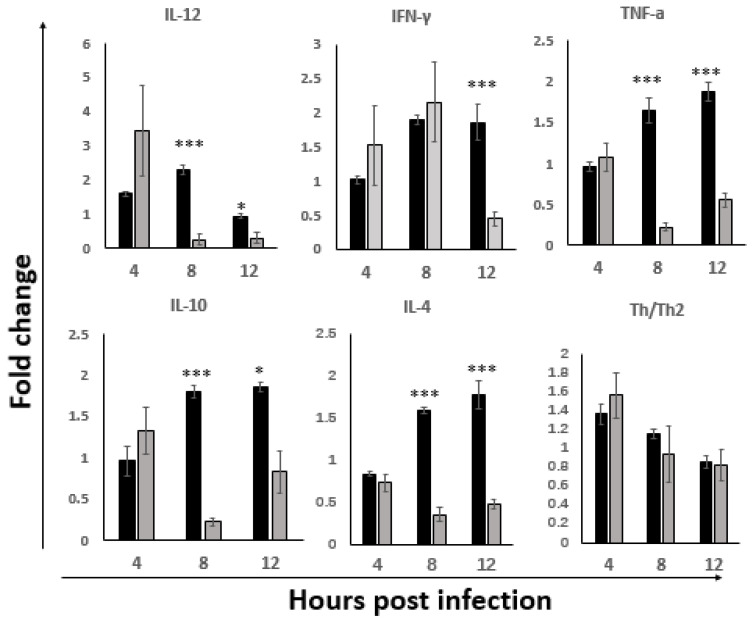
Differential modulations of Th1 and Th2 cytokine response in the ILP cells at the early stage of infection with IBDVs of different virulence. Th1 and Th2 cytokines were detected. Th1/Th2 bias ratio was calculated as average of each Th1/Th2 cytokine. All the data were normalized to uninfected control. Note that the B87 infection downregulated the Th1 and Th2 cytokines at 8 hpi and 12 hpi; NN1172 induced stronger activation of both the Th1 and Th2 cytokines. There was no difference between the B87 and NN1172 groups in Th1/Th2 bias ratio. Results represent mean ± SEM, *n* = 5. (* *p* < 0.05; *** *p* < 0.001) between the HLJ0504-like vvIBDV infected group (black bar) and the attenuated B87 infected group (gray bar).

The cytokines’ response, as showed in Figure 7, demonstrates that the expression trend of both of the Th1/2 cytokines detected in the B87-infected group was downregulated at 8 hpi and 12 hpi, as demonstrated by the fold change value, which were different from the upregulation results in the NN1172 group. However, when the Th1/Th2 bias ratio was calculated, the change trend in the B87 group was similar to that of the NN1172 group, with a stronger Th1 response at 4 hpi and 8 hpi, and then shifted to more of a Th2 bias at 12 hpi. Interestingly, there was no difference between the B87 group and NN1172 group in the Th1/Th2 bias ratio.

These results suggested that vvIBDV induced stronger activation of the macrophage and cytokine response, whereas the attenuated virulent strain suppressed the expression; both HLJ0504-like vv and attenuated virulent IBDV induced a stronger Th1 response in the early infection stage and then shifted to the Th2 bias response.

## 4. Discussion

IBDV is still one of the most important immunosuppressive viral diseases in commercial chickens worldwide because of the evolving of the field strains, as demonstrated by more and more reassortant/novel variant IBDVs being confirmed in recent years [7,34,35], especially the emerging of the novel variant strains with a greatly immunosuppressive effect in the commercial chickens in most of China, which could not be protected by classical vaccines [36,37,38], posing new challenges in the controlling of IBD. Although many efforts have been made to understand the pathogenesis of the virus based on the host-pathogen interaction both in vitro and in vivo [39,40,41], however, most studies have mainly focused on the target tissues such as the BF and the thymus [18]. Little is known about the effects of IBDV on the GALT, the primary site of the viral invasion and infection, and also the first line of defense, especially the response of the innate antiviral response and inflammations. Intestinal mucosa is the first barrier that prevents the invasion of the IBDV after oral route infection. The innate immune response in GALT would determine the disease progression of IBDV infection.

For IBDV infection, the incubation period for clinical signs and mortality is very short, normally about 2 to 3 days post-infection. This period is critical for the immune system of the chicken to respond against the virus, and the innate immune response is the first stage of the response. The chicken TLR3 signaling pathway has been confirmed to be involved in the innate immune response against IBDV and contributed to the pathogenesis in chickens. TLR3 is a trans-membrane protein and is localized to the endosomal compartment which suggests its strong role in the antiviral response [42]. Liu et al. [43] revealed that TLR3- ectodomain (ECDs) binds dsRNA, and the overall shape of the TLR3-ECD does not change upon binding to dsRNA. After IBDV infection, the ECDs of TLR3 could bind to IBDV dsRNA, and the interaction leads to the activation of type I IFN, pro-inflammatory cytokines and chemokines, resulting in the viral elimination or replication in the early infection stage in the BF in the vitro studies [18,44]. In the present study, the expression level of the TLR3-IRF7-IFN-α/β and the downstream antiviral factors PKR, OAS and Mx in ILP cells were significantly upregulated at 8 hpi and 12 hpi as compared to the uninfected control, suggesting that IBDV activates the TLR3 antiviral signal pathway in the gut at the early stage of infection. Since the interplay between IBDV and the antiviral response might have an impact on the replication of the virus, together with the result from Li’s group [9] that there was no detectable IBDV-antigen in the cecum LP even at 3 dpi, we speculated that the early replication of IBDV in the intestinal cells might not be stable, evidenced by the results of the sporadically detected IBDV antigen in ILP cells, which was similar to that of Müller et al. [12].

Interestingly, we noticed that while the activation of TLR3 was continuously increased at 8 hpi and 12 hpi, the expression of IRF7 was downregulated at 12 hpi as compared to that at 8 hpi (Figure 2). IRF3 and IRF7 are two key transcription factors that modulate type I IFN expression upon viral infection [45]. Studies revealed that IRF3 is absent in chickens and other avian species [46]. IRF7 was confirmed to be an important regulator of the antiviral innate immune response in chickens; it could help to determine a chicken’s susceptibility to avian viruses, such as different AIV strains [47]. Ouyang’s group [48] confirmed that the IRF7 signaling pathway played an important role in the IBDV replication in DT40 cells. Together with the disease progression after this early infection period as indicated by the increasing of viral load in BF and the atrophy of BF (Figure 1), we speculated that IRF7 might play an important role in the pathogenesis of IBDV, especially the replication and dissemination of IBDV, which needed to be further explored.

Macrophages are key cellular elements of the innate immune system and play an indispensable role in regulating the interplay between innate and adaptive immune responses. It is reported that viral infections can modulate host immune responses by either enhancing or dampening the effector functions of macrophages, causing immunopathogenesis and/or immunoevasion [49,50]. Additionally, macrophages can also harbor infectious viruses, serving as reservoirs for further dissemination [51]. In the present study, the macrophages were activated at 8 hpi and 12 hpi, suggesting the involvement of macrophages in the pathogenesis of IBDV at the early infection stage, consistent with that of Li et al. [9]. Interestingly, we found that the phenotype of the M1 macrophage was significantly activated at 8 hpi and 12 hpi; however, the iNOS/Arg ratio suggested that the peak activation was at 8 hpi and then shifted to a more non-protective M2 phenotype (Figure 3). Since M1 is involved in destroying microbes while M2 is more important for the tissue-repair and the termination of inflammation, macrophages were proposed to play a critical role in spreading IBDV from the gut to the BF [52]. Together with the peak antiviral signaling response based on the TLR3-IRF7-type I IFN results indicated in Figure 2, we speculated that 8 hpi might be important for viral destiny in the gut-associate lymphocyte in this study: the protective innate immune response before 8 hpi shifted to the non-protective response after 8 hpi, and then continual viral replication afterwards and viral dissemination through the viremia occurred, which was consistent with the progression of the disease.

T cells have been confirmed to play an important role in the clearance of IBDV particles [20,53]. The CD4^+^ helper T (Th) cell, classified as either Th1 or Th2 based on their cytokine profiles [54], play crucial roles. Th1 cells are characterized by the secretion of IFN-γ, IL-2 and IL-12 p40, and evolved to enhance clearance of intracellular pathogens such as viruses [55,56]. Th2 cells are critical for the control of certain parasitic infections through the production of the clustered group of cytokines IL-4, IL-10 and IL-13 [57]. In the present study, both Th1 and Th2 cytokines were upregulated in ILP cells in the early stage of vvIBDV infection. Interestingly, IL-12 was upregulated as early as 4 hpi, and then the upregulation of IFN-γ, IL-12 and IFN-γ are necessary for the development of cytotoxic T cells [58]. Consistently, we also observed the upregulation of TNF-α that is a result from the stimulation of IFN-γ [59]. Furthermore, when the Th1 and Th2 cytokines were compared, a stronger Th1 bias before 8 hpi was observed, and then a shift to the Th2 bias at 12 hpi, consistent with what we observed based on the activation of macrophages and TLR3-IRF7-type I IFN antiviral signaling molecules, suggesting that the cell-mediated responses are initiated to resolve IBDV infections. These results further confirmed that the stronger antiviral response was activated before 8 hpi in ILP cells after the oral infection of IBDV. Though the response might last for a short period as indicated in the present study, it would play an important role in the IBDV replication and dissemination, and the progress of disease. The research results from Li’s group [9] showed that infiltration of T lymphocytes, macrophages and heterophils in the gut, together with the immune-pathogenesis in the gut after 3 dpi of vvIBDV infection, further confirmed that the early interplay of the antiviral response with IBDV affects the progress of disease.

The differential innate immune responses have been confirmed based on the virulence of IBDVs in the bursa or the spleen [17,18,19,44]. However, little is known about the response in the GALT. The present study showed stronger activation of macrophages, an innate antiviral response and more Th cytokine production in the ILP cells in the HLJ0504-like vvIBDV group at 8 hpi and 12 hpi than in the B87 group, consistent with those observed in the bursa by ours and others [18,19], suggesting that the activation of the innate immune response was also stronger by vvIBDV infection in the GALT. Interestingly, we observed that some molecules of the antiviral pathway were stronger activated in the B87 group at 4 hpi, while others were not; however, the activation of macrophages and the Th1/Th2 ratio were not different between the two groups at this time point. Further, as compared to the HLJ0504-like vvIBDV group, the antiviral signaling, macrophage activation and cytokine expression in the B87 group were far more moderated during the early infection stage, consistent with the research from the earlier reports [18,19,60] in the bursa, suggesting that the activation of the antiviral response in the B87 group at 4 hpi might be transient and then downregulated which might be related to the easier dissemination of B87 and consistent with the biology of this vaccine strain.

In conclusion, the present studies provide basic information about the activation of innate antiviral signaling triggered by TLR3, as well as the macrophage activation and cytokine response in the GALT cells after IBDV infection. The results indicated: firstly, the TLR3-IRF7 pathway response, the macrophage activation and the cytokines’ expression in the GALT cells at the early stage of IBDV infection, particularly an earlier period of infection within 12 hr, were differentially upregulated. Secondly, IBDV infection triggered stronger activation of the TLR3-IRF7 pathway at 8 hpi and 12 hpi in the GALT cells. Thirdly, the ILP cells are mainly characterized by the antiviral M1 type and secrete the Th1 cytokines at the time point of 8 hpi, while at the time point of 12 hpi, they seem to be differentiated into the M2 type and exhibited more of a Th2 cytokine response after IBDV infection. Finally, the HLJ-0504-like vvIBDV strain activates a stronger innate antiviral response than the attenuated vaccine strain in the GALT cells. The differential activations at different time points by IBDV and its virulence in the GALT cells might play an important role in the progress of disease. Due to the complexity of the viral pathogenesis and the GALT system of the host as Li et al. [9] were concerned, it is difficult to determine if the gene expression profiles described are from the infected cells or by the bystander cells, since the infected cells and the bystander cells might respond differently. Further, the identity of the exact macrophage is not confirmed, which would present more accurate macrophage profiles. Other factors might also affect the response profiles, such as gut health affected by the microbiota composition, especially the colonization pattern of *Campylobacter jejuni* caused by IBDV infection as demonstrated by Li et al. [9,61]. These have to be evaluated further in the future to improve our understanding of the pathogenesis of IBD.

## Figures and Tables

**Table 1:** The information of the experimental design.

Group	Virus	Hours Post Infection (hpi) ^&^			Days Post Infection (dpi) ^&^			
		4	8	12	1	3	5	7
1	NN1172	IH ^@^, innate antiviral profiles of the ILP cells ^$^, viral load			BBIX and Bursal viral load			
2	B87	innate antiviral profiles of the ILP cells ^$^, viral load			-			
3	PBS	IH ^@^, innate antiviral profiles of the ILP cells ^$^, viral load			BBIX and Bursal viral load			

^&^ 5 birds at each time point per group. ^@^ Immunohistochemistry (IH). ^$^ TLR3, IRF7, IFN-α/β, PKR, OAS, Mx, iNOS, Arg, IL-10, IL-12, IFN-γ, IL-4, TNF-α, GAPDH.

**Table 2:** Sequences of primers used for quantitative reverse transcription-polymerase chain reaction for detection of chicken and IBDV genes of interest.

Genes	Direction	Sequence	Product (bp)	Accession no. in GenBank
TLR3 [18]	Forward	GCAACACTTCATTGAATAGCCTTGAT	92	NM001011691.3
	Reverse	GCCAAACAGATTTCCAATTGCATGT		
IRF7 [25]	Forward	ACCACATGCA GACAGACTGACACT	146	AF268079
	Reverse	GGAGTGGATGCAAATGCTGCTCTT		
IFN-α [26]	Forward	GGACATGGCTCCCACACTAC	75	GU119896.1
	Reverse	TCCAGGATGGTGTCGTTGAAG		
IFN-β [18]	Forward	TTCTCCTGCAACCATCTTC	82	NM001024836.1
	Reverse	GAGGTGGAGCCGTATTCT		
PKR [27]	Forward	CCTCTGCTGGCCTTACTGTCA	151	AB125660.1
	Reverse	AAGAGAGGCAGAAGGAATAATTTGCC		
OAS [28]	Forward	CACGGCCTCTTCTACGACA	103	AB037592.1
	Reverse	TGGGCCATACGGTGTAGACT		
Mx [25]	Forward	TTCACGTCAATGTCCCAGCTTTGC	85	NM204609
	Reverse	ATTGCTCAGGCGTTTACTTGCTCC		
iNOS	Forward	CAGCCAGCTCATCCGATAT	307	D85422.1
	Reverse	TTCCAGACCTCCCACCTC		
Arg	Forward	CTTGGCACTTGGTTCTGT	169	NM001199704.1
	Reverse	GCTGTGGGACTTTATCTTG		
IL-10 [26]	Forward	ATGCTGCGCTTCTACACA	73	NM001004414.2
	Reverse	CCATGCTCTGCTGATGACT		
IL-12 [26]	Forward	TCTGCTAAGACCCACGAGA	82	DQ202328.1
	Reverse	TTGACCGTATCATTTGCCCAT		
IFN-γ [29]	Forward	CTGAAGAACTGGACAGAGAG	264	NM205149.2
	Reverse	CACCAGCTTCTGTAAGATGC		
IL-4 [30]	Forward	TGTGCTTACAGCTCTCAGTG	212	NM001007079.1
	Reverse	ACGCATGTTGAGGAAGAG		
TNF-α	Forward	TTGCCCTTCCTGTAACCA	66	HQ739087.1
	Reverse	AGCCAAGTCA ACGCTCCT		
GAPDH [18]	Forward	GCCATCACAGCCACACAGA	120	NM204305.2
	Reverse	TTTCCCCACAGCCTTAGCA		
IBDV-VP2 [31]	Forward	ACCGGCACCGACAACCTTA	117	FJ615511.1
	Reverse	CCCTGCCTGACCACCACTT		

## Data Availability

Data is contained within the article or supplementary material. The data presented in this study are available in insert article or supplementary material here.

## Supplementary Materials

The following are available online at https://www.mdpi.com/article/10.3390/v14020393/s1, Table S1. The standard curve, R2 and amplification sufficiency of RT-qPDR for iNOS, Arg, and TNF-α.
