# Supplementary material for: Differential Modulation of Innate Antiviral Profiles in the Intestinal Lamina Propria Cells of Chickens Infected with Infectious Bursal Disease Viruses of Different Virulence
Source: Viruses. 2022 Feb 15;14(2):393. doi: 10.3390/v14020393 (PMC8878311; doi:10.3390/v14020393)
Supplement: Supplementary file 1 [file viruses-14-00393-s001.zip › viruses-1543774-supplementary.pdf]

Supplementary Table S1. The standard curve, R2 and amplification sufficiency of RT-qPCR for iNOS, Arg, and TNF-α

Gene	Standard curve	R^2 of calibration curve	Amplification efficiency (E)
iNOS	Y=-3.334x+36.548	0.999	99.685%
Arg	Y=-3.330+37.204	0.999	99.685%
TNF-α	Y=-3.368+38.573	0.998	98.101%
